# Nonlinear relationship between serum ferritin and diabetic retinopathy in patients with type 2 diabetes mellitus: mediating role of neutrophil-to-albumin ratio

**DOI:** 10.3389/fendo.2026.1803600

**Published:** 2026-04-29

**Authors:** Xiaoyu Lv, Binjing Pan, Yirong Wang, Xinyuan Guo, Jingfang Liu

**Affiliations:** 1 The First Clinical Medical College, Lanzhou University, Lanzhou, Gansu, China; 2 Department of Endocrinology, The First Hospital of Lanzhou University, Lanzhou, Gansu, China

**Keywords:** diabetes mellitus, diabetes retinopathy, ferritin, neutrophil-to-albumin ratio, T2DM

## Abstract

**Aims:**

To clarify the relationship between serum ferritin (SF) levels and diabetic retinopathy (DR) and to analyze the mediating role of the neutrophil-to-albumin ratio (NAR).

**Methods:**

A total of 1,884 middle-aged and elderly patients (aged ≥ 45 years) with type 2 diabetes mellitus (T2DM) were included in this study. Participants were divided into non-DR and DR groups. Adjusted regression models, receiver operating characteristic (ROC) curve analysis, threshold effect analysis, and mediation effect analysis were performed. T2DM patients were divided into three groups, and differences in the prevalence of DR were compared.

**Results:**

SF and NAR levels were higher in the DR group than in the non-DR group (P < 0.05). Logistic regression analysis showed a significant positive correlation between SF levels and the risk of DR. Restricted cubic spline (RCS) and threshold effect analyses revealed a nonlinear relationship between SF and the risk of DR, with an optimal inflection point at a SF value of 27.9556. Additionally, NAR was positively correlated with the risk of DR. The prevalence of DR increased with increasing levels of NAR markers and SF (*P* < 0.05). Mediation analysis of the association between NAR and the risk of DR showed that the total effect estimate was 0.0003, the direct effect estimate was 0.0003, and the mediation effect estimate was 0.0001, with the mediation proportion accounting for 20.99% of the total effect. The combination of SF and NAR demonstrated potential as an indicator for DR, with an area under the ROC curve (AUC) of 0.69.

**Conclusions:**

SF levels in middle-aged and elderly patients with T2DM are associated with an increased risk of DR. NAR, an inflammatory marker, plays an important mediating role in the association between ferritin and the risk of DR. The combined use of SF and NAR may serve as a more sensitive indicator for the early diagnosis of DR.

## Introduction

The prevalence of diabetes has rapidly increased worldwide due to changes in dietary patterns. According to data from the International Diabetes Federation, the number of people aged 20–79 with diabetes is projected to reach 642 million worldwide by 2040 (1). The most serious harm caused by diabetes mainly lies in the various complications it triggers. DR is a microvascular complication and one of the leading causes of blindness in adults.

Because advanced DR can severely damage vision and lead to irreversible blindness, timely prevention and treatment are crucial. However, current treatment methods, such as lipid reduction, laser photocoagulation, and vitrectomy, mostly target advanced stages of the disease and carry certain side effects and risks to prognosis. Therefore, there is an urgent need to investigate the early molecular mechanisms and potential treatment of this disease. Currently, the control of DR risk factors mainly focuses on managing blood glucose, blood lipids, blood pressure, and unhealthy lifestyle habits (such as smoking). However, research on other contributing factors remains limited. Studies have shown that vitamin D levels, iron homeostasis, visceral fat, insulin resistance, and obesity influence the occurrence and progression of DR in different ways (2–7).

SF is an important iron-storage protein in the human body that reflects nutritional status and iron reserves. Studies have demonstrated that elevated SF levels are associated with obesity, diabetes, and metabolic syndrome (8, 9). Many studies have also indicated that iron overload and ferroptosis contribute to the microvascular lesions of DR through various mechanisms, including oxidative stress, mitochondrial dysfunction, endoplasmic reticulum stress, and inflammatory responses. Therefore, to further clarify the association between SF levels and DR, and to investigate whether chronic inflammation plays a potential mediating role in this process, this study analyzed the relationship between SF levels and the risk of DR in middle-aged and elderly patients with type 2 diabetes mellitus (aged ≥ 45 years), with the aim of providing new predictive indicators for the early diagnosis of DR.

## Study population and methods

### Study population

**Figure 1:**
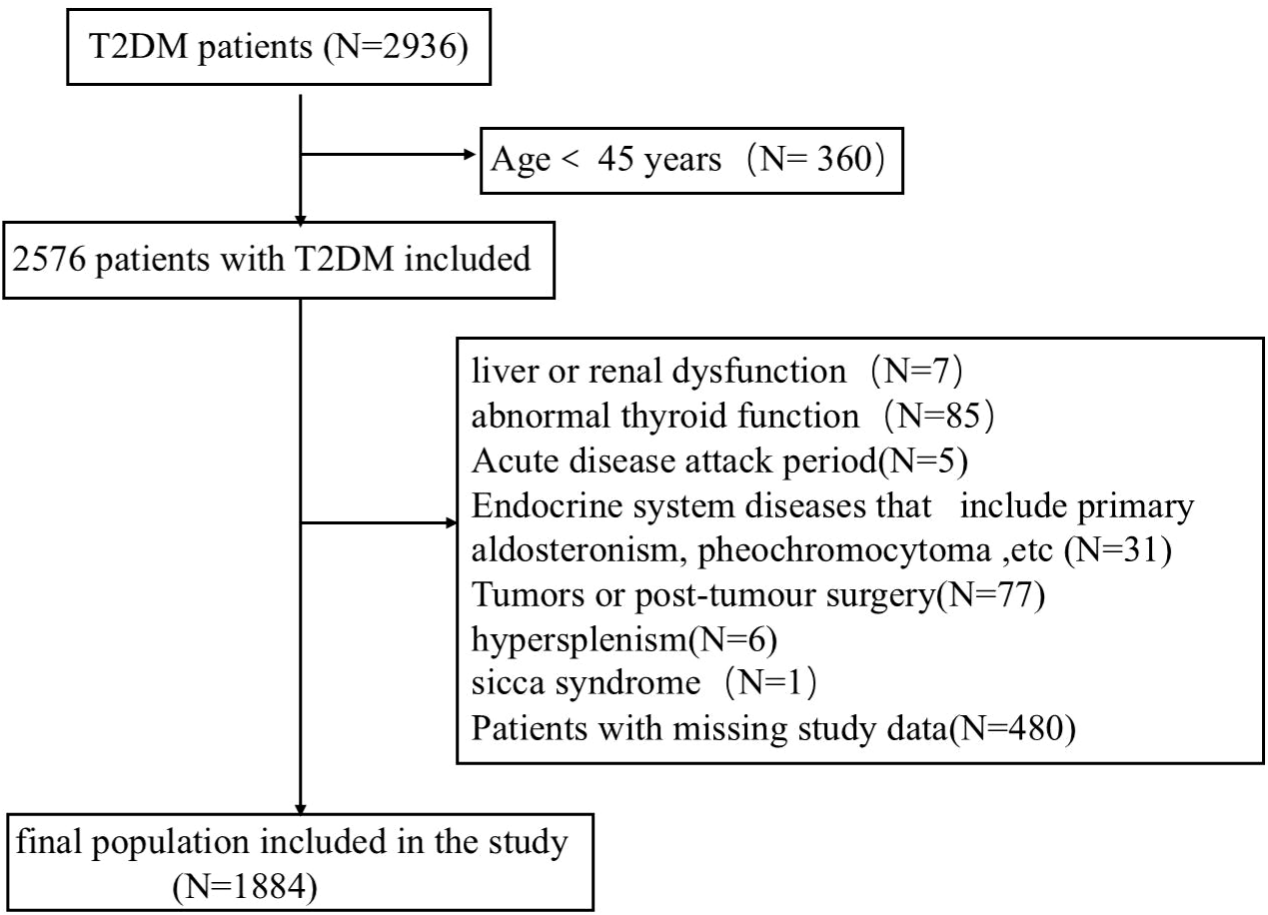
Screening of research subjects.

The research subjects included 2,084 patients with type 2 diabetes who were hospitalized at the Endocrinology Department of Lanzhou University First Hospital from August 2016 to March 2024. After screening according to the inclusion and exclusion criteria, 1,884 patients with type 2 diabetes were finally included (**Figure 1**). A flowchart of the participant screening process is shown in **Figure 1**.

### Inclusion criteria

(1) All participants were adults aged ≥ 45 years (2); the diagnosis of T2DM was based on the Chinese Guidelines for the Prevention and Treatment of T2DM (2020 edition) (10), with a disease duration of ≥1 year (3); study data were complete.

### Exclusion criteria

(1) Patients with type 1 diabetes or other specific types of diabetes (including adult-onset autoimmune diabetes and gestational diabetes); (2) patients with a history of malignant tumors or tumor resection; (3) patients with acute or chronic liver or kidney dysfunction; (4) patients with other endocrine-related diseases, including pituitary tumors, prolactinomas, or primary aldosteronism; (5) patients with acute diabetic complications or severe heart failure; (6) patients with acute or chronic infectious diseases;(7) patients with tumor, or diseases affecting the hematological system; and (8) patient with insufficient relevant information.

### Collection of clinical data

After all participants had fasted for 8 hours, venous blood samples were collected in the early morning of the following day. The levels of albumin(ALB),total bilirubin (TBIL), direct bilirubin (DBIL), triglycerides (TG), total cholesterol (TC), high-density lipoprotein cholesterol (HDL-C), low-density lipoprotein cholesterol (LDL-C), alanine aminotransferase (ALT), aspartate aminotransferase (AST), γ-glutamyl transferase (GGT), fasting plasma glucose (FPG), and blood uric acid (UA) were measured using a BS-220 automatic biochemical analyzer. Glycated hemoglobin (HbA1c) was measured using high-performance liquid chromatography (Bio-Rad Variant II Glycosylated Hemoglobin Detection Automatic Analyzer). Fasting insulin (FINS) levels were determined using a chemiluminescence immunoassay (CENTAUR-XP automated chemiluminescence immunoanalyzer; Siemens Healthineers). Serum 1,25-dihydroxyvitamin D3(25(OH)D) levels were determined using an RT-6000 enzyme analyzer (Shenzhen Leidu Life Science Co., Ltd.).The homeostasis model assessment of insulin resistance (HOMA-IR) was calculated as follows: HOMA-IR = FPG (mmol/L) × FINS (mU/L)/22.5. SF levels were determined using an electrochemiluminescence immunoassay (Roche, Shanghai, China). Neutrophil counts (NEUT) were obtained using a Mindray automated blood cell analyzer. Neutrophil-to-albumin ratio (NAR) was calculated as NEUT/ALB.

### The diagnostic criteria for DR

Professional ophthalmologists conduct fundus photography of patients, and the diagnosis of DR was based on international clinical grading criteria and adheres to current domestic diagnostic and treatment guidelines as well as expert consensus (11, 12).

### Study grouping

All patients with T2DM were divided into DR and non-DR groups.

Based on the tertile levels of NAR and ferritin, the T2DM patients were divided into three groups, and differences in the prevalence of DR were compared.

Based on the threshold effect, the T2DM patients were divided into two groups to compare differences in the prevalence of DR.

### Data analysis

All data were analyzed using IBM SPSS 26.0 and R 4.4.1. The measurement data following a normal distribution were expressed as mean ± standard deviation (x̄ ± s), and the inter-group comparison was conducted using a t-test. For non-normal data, the median (P25, P75) was used to represent them, and non-parametric tests were employed for inter-group comparisons. The count data were presented as frequency and percentage (%), and the chi-square test was used for intergroup analysis. Multiple comparisons were adjusted for significance using the Bonferroni correction. Univariate logistic regression was used to analyze the associations between DR and ferritin and NAR levels. Multivariate logistic regression was used to analyze the independent associations between DR, Ferritin, and NAR levels. The threshold effect and restricted cubic spline (RCS) curve analyses were used to analyze the nonlinear relationship between DR and SF levels. Receiver operating characteristic (ROC) curve analysis was used to evaluate the predictive value of SF and the combined diagnosis of SF and NAR for the risk of DR occurrence. Mediator effect analysis was conducted to explore the potential mediating role of NAR in the relationship between SF levels and the risk of DR. All tests were two-sided, and a *p* value < 0.05 was considered statistically significant.

## Results

### Comparison of general data between the DR and the Non-DR groups

Compared with the non-DR group, age, systolic blood pressure (SBP), prevalence of hypertension, duration of diabetes, HbA1c, FPG, FINS, HOMA-IR, ALT, TBIL, TG, SF, UA, homocysteine (HCY), and NAR were higher in the DR group, whereas calcium (Ca) and 25(OH)D levels were lower (all *p* < 0.05). There were no statistically significant differences in the other indicators between the two groups (all *p*>0.05) (**Table 1**).

**Table 1:** Baseline characteristics in the DR and non-DR groups.

Variables	Non-DR (n = 1093)	DR (n = 791)	*P*
Age(year)	60.64 ± 8.47	63.69 ± 8.30	<0.001
Gender			
Male(n,%)	703 (64.38)	531 (67.13)	0.215
Female(n,%)	389(35.62)	260 (32.87)	
BMI(kg/m^2^)	24.04 ± 3.48	24.01 ± 3.08	0.817
Smoking(n,%)	223 (20.40)	163 (20.61)	0.914
Drinking(n,%)	146(13.36)	97 (12.26)	0.484
SBP(mmHg)	141.95 ± 24.77	147.38 ± 23.00	<0.001
DBP(mmHg)	85.27 ± 15.75	84.68 ± 15.08	0.415
Hypertension(n,%)	561(51.33)	466(58.91)	0.001
diabetes duration(year)	8.00 (3.00, 14.00)	11.00 (6.00, 18.00)	<0.001
HbA1c(%)	8.47 ± 2.12	8.79 ± 2.03	<0.001
FPG(mmol/L)	7.88 (6.52, 10.22)	8.73 (6.91, 11.01)	<0.001
FCP(mIU/L)	1.24 (0.88, 1.69)	1.25 (0.90, 1.80)	0.139
FINS(ng/Ml)	6.18 (4.25, 9.26)	6.74 (4.29, 10.68)	0.018
HOMA-IR	2.30 (1.45, 3.53)	2.55 (1.56, 4.46)	<0.001
AST(U/L)	19.00 (15.00, 25.00)	19.00 (16.00, 23.00)	0.580
ALT(U/L)	19.00 (15.00, 30.00)	20.30 (15.30, 28.30)	0.005
TBil(μmol/L)	14.60 (11.40, 18.70)	15.05 (11.95, 19.00)	0.031
DBil(μmol/L)	2.70 (2.10, 3.60)	2.80 (2.20, 3.60)	0.141
Ca(mmol/L)	2.18 ± 0.14	2.16 ± 0.14	0.040
TC(mmol/L)	4.11 (3.37, 4.77)	4.13 (3.44, 4.88)	0.125
TG(mmol/L)	1.51 (1.08, 2.19)	1.67 (1.27, 2.27)	<0.001
HDL(mmol/L)	1.06 ± 0.28	1.05 ± 0.24	0.585
LDL(mmol/L)	2.69 ± 0.81	2.75 ± 0.83	0.135
25(OH)D(ng/mL)	13.40 (9.70, 17.70)	11.30 (7.80, 15.85)	<0.001
SF(μg/L)	121.00 (59.20, 242.00)	151.00 (93.50, 279.00)	<0.001
UA(μmol/L)	320.00 (262.00, 377.00)	327.00 (272.50, 381.00)	<0.001
HCY(μmol/L)	13.30 (10.30, 17.20)	14.50 (11.50, 19.20)	<0.001
NAR	0.68 (0.55, 0.87)	0.84 (0.71, 1.08)	<0.001

BMI, body mass index; SBP, systolic blood pressure; DBP, diastolic blood pressure; FPG, fasting glucose; FCP, fasting C peptide; FINS, fasting insulin; HOMA-IR, Homeostasis model assessment of insulin resistance; AST, aspartate aminotransferase; ALT, alanine aminotransferase; TBIL, Total Bilirubin, TBIL; DBIL, Total Bilirubin, TBIL; GGT, γ-glutamyl transferase; TC, Total Cholesterol; HDL, high density lipoprotein; LDL, low density lipoprotein; HCY, homocysteine; TG, triglyceride; UA, uric acid; NAR, neutrophil-to-albumin.

### Single-factor logistic regression analysis of the influence of SF on the risk of DR

DR was considered the dependent variable, and age, SBP, prevalence of hypertension, duration of diabetes, HbA1c, FPG, FINS, HOMA-IR, ALT, TBIL, TG, UA, HCY, Ca, 25(OH)D, SF, and NAR were used as independent variables. Single-factor logistic regression analysis showed that Age, SBP, prevalence of hypertension, duration of diabetes, HbA1c, FPG, HOMA-IR, TG, UA, HCY, SF, and NAR were positively associated with the risk of DR, whereas Ca and 25(OH)D were negatively associated with the risk of DR (all *p* < 0.05). FINS and ALT levels were not significantly associated with DR risk (all *p*>0.05) (**Table 2**).

**Table 2:** Logistic regression analysis of DR and study indicators levels.

Variables	β	*P*	OR(95%CI)
Age(year)	0.0428	<0.001	1.0437 (1.0322 ~ 1.0553)
SBP(mmHg)	0.0093	<0.001	1.0094 (1.0055 ~ 1.0133)
Hypertension(n,%)	0.3073	0.0011	1.3597 (1.1304 ~ 1.6356)
diabetes duration(year)	0.0601	<0.001	1.0620 (1.0483 ~ 1.0758)
HbA1c(%)	0.0739	0.0010	1.0767 (1.0304 ~ 1.1250)
FPG(mmol/L)	0.0641	<0.001	1.0662 (1.0366 ~ 1.0967)
FINS(ng/mL)	0.0118	0.0544	1.0119 (0.9998 ~ 1.0242)
HOMA-IR	0.0365	0.0086	1.0372 (1.0093 ~ 1.0659)
ALT (U/L)	0.0053	0.0743	1.0053 (0.9995 ~ 1.0111)
TBil(μmol/L)	0.0100	0.1806	1.0101 (0.9954 ~ 1.0250)
TG(mmol/L)	0.0565	0.0470	1.0581 (1.0008 ~ 1.1188)
UA(μmol/L)	0.0012	0.0196	1.0012 (1.0002 ~ 1.0023)
HCY(μmol/L)	0.0323	<0.001	1.0329 (1.0185 ~ 1.0475)
Ca(mmol/L)	-0.7040	0.0418	0.4946 (0.2511 ~ 0.9743)
25(OH)D(ng/mL)	-0.0483	<0.001	0.9528 (0.9376 ~ 0.9683)
SF(μg/L)	0.0013	<0.001	1.0013 (1.0007 ~ 1.0018)
NAR	1.7822	<0.001	5.9430 (4.3245 ~ 8.1673)

SBP, systolic blood pressure; DBP, diastolic blood pressure; FPG, fasting glucose; FCP, fasting C peptide; FINS, fasting insulin; HOMA-IR, Homeostasis model assessment of insulin resistance; ALT, alanine aminotransferase; TBIL, Total Bilirubin, TBIL; HCY, homocysteine; TG, triglyceride; UA, uric acid; NAR, neutrophil-to-albumin.

### Multivariate logistic regression analysis of the relationship between each indicator and the risk of DR

Based on the results of the above differential and single-factor logistic regression analyses, DR was used as the dependent variable, and a multivariate logistic regression analysis was conducted to examine the effects of ferritin and the inflammatory indicator NAR on DR. In Model 1, no variables were adjusted. The results showed that ferritin levels were independently and positively associated with the risk of DR (*p* < 0.05). In Model 2, after adjusting for age and duration of diabetes, SF levels remained independently and positively associated with the risk of DR (*p* < 0.05). In Model 3, after adjusting for age, duration of diabetes, SBP, HbA1c, FPG, FINS, HOMA-IR, ALT, TBil, Ca, 25(OH)D, prevalence of hypertension, UA, TG, HCY, ferritin, and NAR were independently and positively associated with the risk of DR (both *p* < 0.05) (**Table 3**).

**Table 3:** Logistic regression analysis of DR and some indicators levels.

Index	Variables	β	*P*	OR(95%CI)
Model1	SF	0.0013	<0.001	1.0013 (1.0007 ~ 1.0018)
Model2	SF	0.0019	<0.001	1.0019 (1.0014 ~ 1.0025)
Model3	SF	0.0014	<0.001	1.0014 (1.0008 ~ 1.0020)
	NAR	1.5136	<0.001	4.5429 (3.2152 ~ 6.4187)

Model 1: Unadjusted variable; Model 2: Adjusted Age, diabetes duration; Model 3: Adjusted Age, diabetes duration, SBP, HbA1c, FPG, FINS, HOMA-IR, ALT, TBil, Ca, 25(OH)D, Hypertension, UA, TG, HCY.

### Analysis of the nonlinear relationship between SF and the risk of DR through threshold analysis

**Figure 2:**
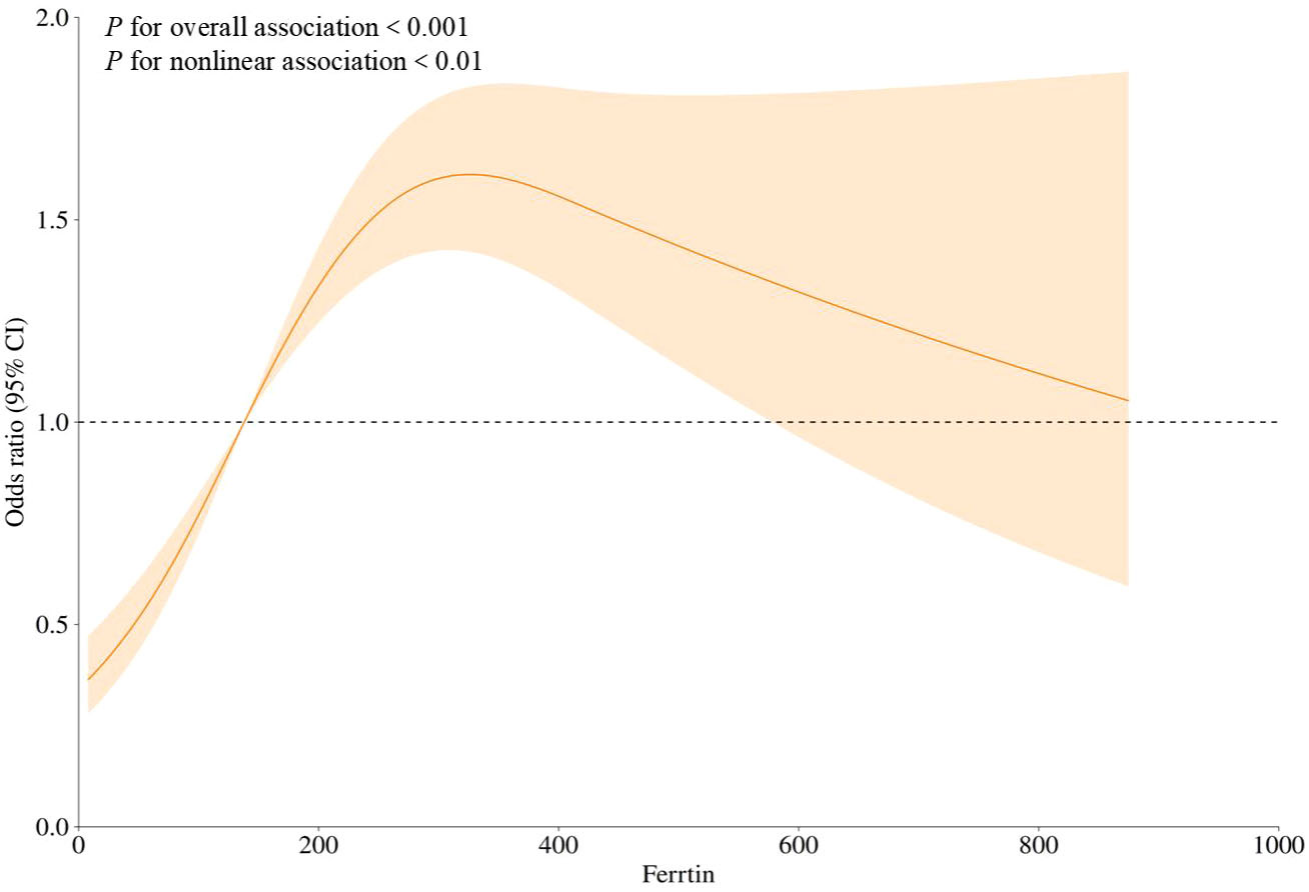
Threshold effect exploring the nonlinear relationship between SF and the risk of DR. NAR, neutrophil-to-albumin; DR,diabetic retinopathy.

SF level was used as the independent variable, and the presence or absence of DR was used as the dependent variable. Age, duration of diabetes, SBP, HbA1c, FPG, FINS, HOMA-IR, ALT, TBil, Ca, and 25(OH)D levels, as well as prevalence of hypertension, UA, TG, and HCY, were included as covariates. Threshold analysis with variable adjustments was conducted. The results showed a significant threshold effect on the association between SF levels and the risk of DR (*p* for likelihood test < 0.001). Overall, SF levels were positively associated with the risk of DR [OR (95% CI):1.0017 (1.0011–1.0023)]. When the SF level was < 27.9556 ng/dL, no association was found between SF levels and risk of DR. However, when the SF level was greater than 27.9556 ng/dL, it was positively associated with the risk of DR [OR (95% CI): 1.0010 (1.0004–1.0017)] (**Table 4**). Additionally, the RCS curve confirmed a nonlinear relationship between SF levels and the occurrence of DR (**Figure 2**).

**Table 4:** Threshold analysis between DR and SF indexes.

Outcome	Effect	*P*
Model 1 Fitting model by standard linear regression Model 2 Fitting model by two-piecewise linear regression	1.0017 (1.0011 - 1.0023)	<0.001
Inflection point	27.9556	
<27.9556	1.0000 (0.0000 -Inf)	1.0000
≥27.9556	1.0010 (1.0004- 1.0017)	<0.001
*P* for likelihood test		<0.001

Adjusted Age, diabetes duration, SBP, HbA1c, FPG, FINS, HOMA-IR, ALT, TBil, Ca, 25(OH)D, Hypertension, UA, TG, HCY.

### Comparison of DR prevalence among different SF levels and NAR level groups

**Figure 3:**
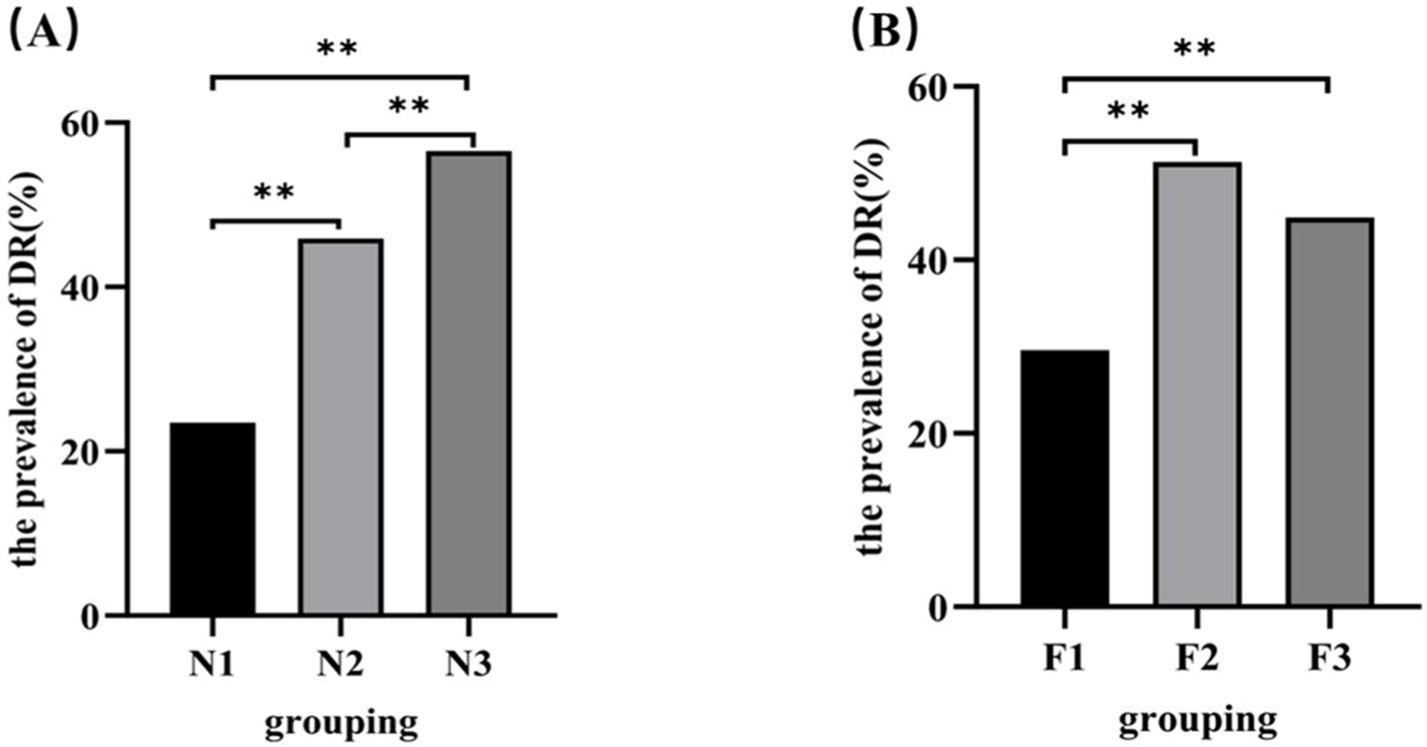
Comparison of the prevalence of DR based on different levels of NAR and SF. NAR, neutrophil-to-albumin; **(A)** Prevalence of DR at different NAR tertile levels; **(B)** Prevalence of DR at different ferritin tertile levels. **p<0.001.

After grouping participants based on tertiles of NAR (N1–N3 groups), the results showed that the prevalence of DR in the N2 and N3 groups (45.85% and 56.51%, respectively) was higher than that in the N1 group (23.47%, both *p* < 0.05), and the prevalence of DR in the N3 group was higher than that in the N2 group (*p* < 0.05) (**Figure 3A**).

The groups were divided based on the tertile of SF levels. The results indicated that the prevalence of DR in the F2 and F3 group (51.25% and 44.85%, respectively) was significantly higher than that in the F1 group (29.58%, both *p* < 0.05), and there was no significant difference in the prevalence of DR between the F2 group and F3 group (*p*> 0.05) (**Figure 3B**).

### Mediation analysis

**Figure 4:**
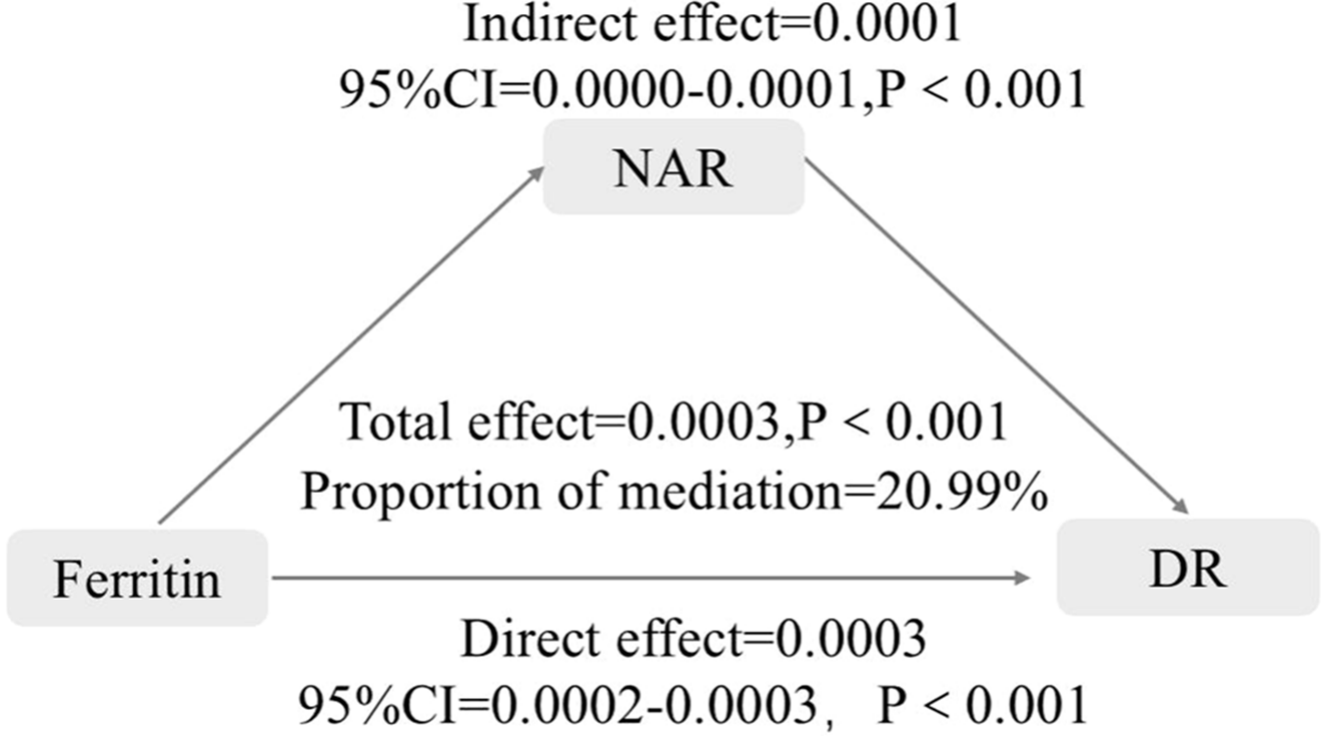
Mediation analysis between SF and the risk of DR. NAR, neutrophil-to-albumin.

To investigate whether the inflammatory marker NAR plays a role in the relationship between ferritin levels and the risk of DR, with the presence or absence of DR as the dependent variable, the SF level as the independent variable, age, duration of diabetes, SBP, HbA1c, FPG, FINS, HOMA-IR, ALT, TBil, Ca, 25(OH)D, prevalence of hypertension, UA, TG, HCY as covariates, and NAR as the mediator variable. The results indicated that the SF level significantly affected the risk of DR (β=0.0003), and NAR has a partial mediating effect in the relationship between the SF level and the risk of DR, with a mediating effect size of 20.99% (β=0.0001) (**Figure 4**).

### Analysis of the predictive value of NAR and SF levels for the risk of developing DR

Using SF and the combination of SF and NAR as test variables, and whether T2DM patients had DR as the status variable (1 for yes, 0 for no), the critical value for the occurrence of DR in T2DM patients was obtained using the ROC curve.

**Figure 5:**
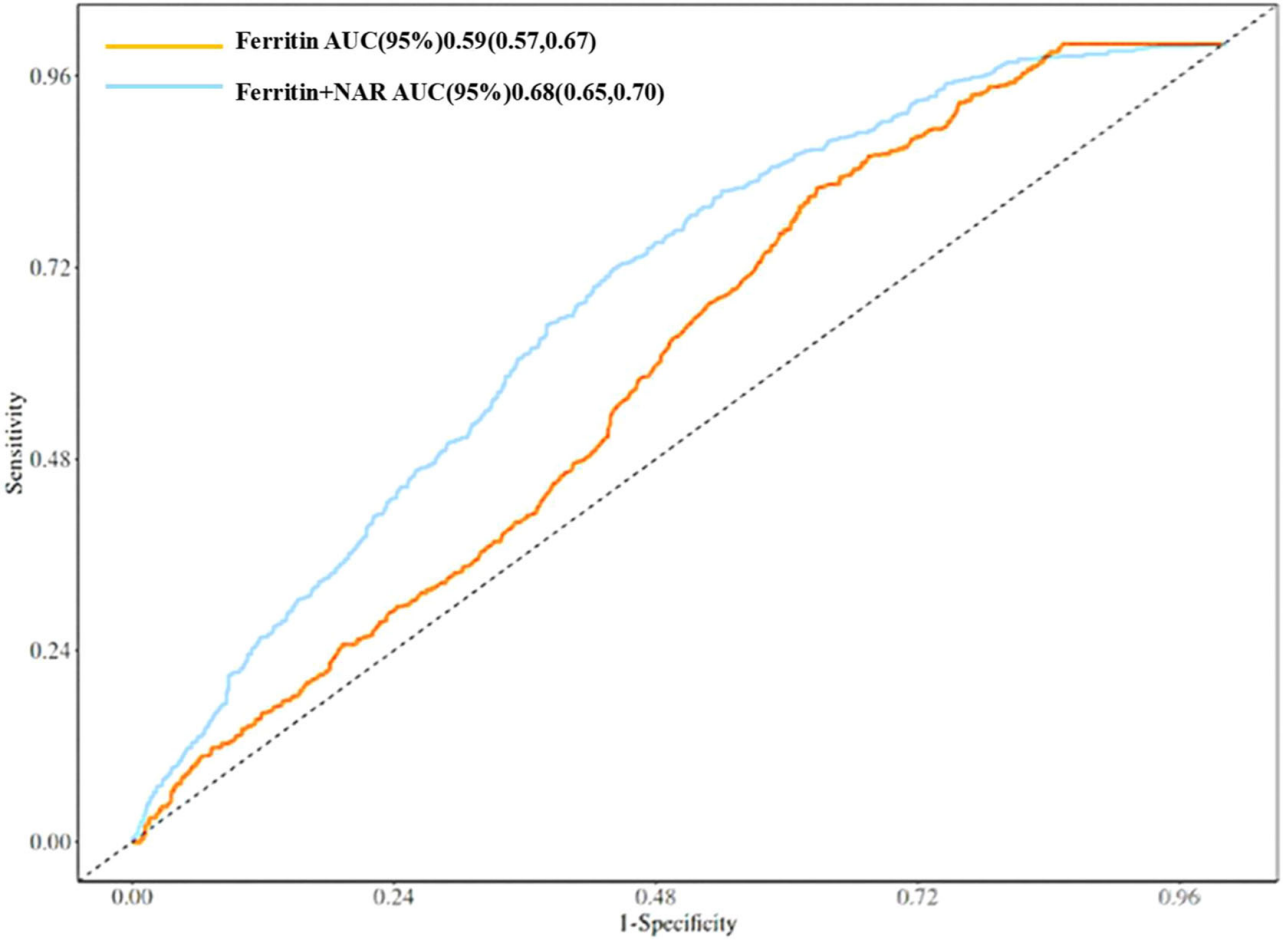
Predictive analysis of the risk of DR by NAR and SF. NAR, neutrophil-to-albumin.

The results showed that SF was used as the test variable, the area under the curve was 0.59, *p* < 0.05, 95% CI (0.57, 0.62), the cut-off value of SF was 79.85, the sensitivity was 0.37, and the specificity was 0.82. However, SF+NAR were combined as the test variable; the area under the curve was 0.68, *p* < 0.05, 95% CI (0.65, 0.70). The cutoff value for the combined indicator was 0.375,sensitivity was 0.56,and specificity was 0.72; Therefore, the diagnostic value of the combined marker SF+NAR is higher than that of SF alone for Diabetic Retinopathy. (**Figure 5**).

## Discussion

SF is widely recognized as an indicator of total body iron stores. Elevated SF levels indicate iron overload, which is associated with the production of reactive oxygen species (ROS) (13). Additionally, Recalcati et al. found that hyperferritinemia is associated with proinflammatory and immunosuppressive effects (14).

The present study showed that SF levels were positively associated with the risk of DR, and this association remained significant after adjusting for multiple confounding factors. A nonlinear relationship was observed between SF levels and the DR risk, with a turning point at a SF level of 27.9556 mg/dL. On the left side of the inflection point, the SF levels were not associated with DR. Conversely, on the right side of the inflection point, the SF levels is positively associated with the risk of DR. Based on the threshold effect analysis, patients were grouped according to the identified inflection point, and it was demonstrated that when SF levels were ≥ 27.9556 mg/dL, the prevalence of DR is positively associated with the SF levels. Regression analysis demonstrated that SF levels were positively associated with the risk of DR [OR (95% CI): 1.0017 (1.0011–1.0023)]. However, the OR was relatively small. At the level of the individual patient, the clinical utility of monitoring SF levels alone for predicting or diagnosing DR is likely to be very limited. It is therefore unlikely to serve as a standalone clinical decision-making tool. Nevertheless, the observed statistical significance indicates a genuine, albeit modest, association between SF levels and the occurrence of DR. This indicates that the development of DR is a complex process involving multifactorial pathophysiological mechanisms, with potentially additional influencing factors participating. More in-depth mechanistic studies and longitudinal data are needed to clarify its precise role in the pathogenesis and progression of DR. Additionally, this study highlights the role of the inflammatory index NAR in moderating the relationship between SF levels and the risk of DR occurrence, with NAR accounting for 20.99% of the effect of ferritin on DR. These findings not only confirm the association between SF and DR but also emphasize the multifaceted involvement of inflammatory responses in DR.

Smotra et al. (15) reported a significant association between elevated SF levels and diabetic complications, including nephropathy, retinopathy, and neuropathy. A study from a Turkish ophthalmology center serum iron status markers, especially the increase in ferritin level, may be a potential biomarker for diabetic macular oedema (16). Current research indicates that iron overload and ferroptosis are extensively involved in the development of microvascular complications in DR through various mechanisms, including oxidative stress, mitochondrial dysfunction, endoplasmic reticulum stress, and inflammatory responses. Increased iron accumulation in the retina can enhance hydroxyl radical production and oxidative stress through the Fenton/Haber-Weiss reaction, thereby promoting lipid peroxidation and iron death, which in turn damages retinal pigment epithelial cells, retinal endothelial cells, and neurons (17, 18). The extent of damage caused by iron overload-induced oxidative stress varies among different retinal cell types, leading to changes in SF levels.

Misra and Liu J et al. supported this association by describing several iron overload mouse models that developed retinal lesions similar to those observed in DR (19, 20). Additionally, other studies have demonstrated that iron overload in diabetic mice exacerbates the progression of DR (21). Furthermore, 20-week-old db/db mice exhibited systemic iron overload and increased ferritin expression in the retina, consistent with the findings of Chaudhary et al. (22) in 2018. Since ferritin expression is directly proportional to iron levels in systemic tissues, most studies have used ferritin expression as an indirect indicator of retinal iron content (23), demonstrating that increased ferritin expression in the retina reflects retinal iron overload.

Mitochondrial dysfunction is a key feature of diabetes (24) and a primary trigger of oxidative stress in endothelial cells (25). Furthermore, mitochondrial dysfunction disrupts intracellular iron balance, while iron overload promotes oxidative stress and membrane lipid peroxidation, interferes with adenosine triphosphate (ATP) synthesis, and exacerbates the permeability of the mitochondrial inner and outer membranes. This vicious cycle ultimately leads to increased retinal oxidative stress and an inflammatory cytokine storm (25, 26). Iron is inextricably linked to retinal oxidative stress and high levels of inflammation through mitochondrial dysfunction. These findings are consistent with our results; however, some researchers have reported in a case-control study that DR is not significantly associated with serum iron or ferritin levels (27). This was consistent with the findings of Elis et al. (28).

In addition, ferritin can also function as an acute-phase reactant. As an acute-phase protein, its levels may also rise under inflammatory conditions (29, 30). Additionally, ferritin can disrupt glucose metabolism through systemic inflammatory responses, thereby increasing the risk of DR (31).Acting as an acute-phase reactant, ferritin plays a pivotal role in maintaining iron homeostasis during inflammation, which is crucial for host defense against infections, tissue damage, and cancer (32). The synthesis of ferritin is fine-tuned at both transcriptional and translational levels by inflammatory signals and oxidative stress (14). Specifically, the pro-inflammatory cytokine tumor necrosis factor-alpha upregulates ferritin H-chain expression via an iron regulatory protein/iron-responsive element-independent mechanism that relies on nuclear factor kappa B activity (33). Similarly, interleukin-1β enhances the translation of H-ferritin mRNA through direct interaction with its 5’ untranslated region (34). Thus, ferritin serves not only as a key acute-phase responder but also as an amplifier of inflammatory cascades. Chronic inflammatory responses over the long term also accelerate the progression of diabetic microvascular complications (35–37). The diabetic microenvironment activates both local and systemic inflammatory responses, thereby promoting the activation of numerous inflammatory cells (38, 39). A chronic inflammatory microenvironment leads to microvascular endothelial cell damage and apoptosis, further exacerbating diabetic microvascular complications (40). Moreover, inflammation can trigger iron metabolism dysregulation by affecting regulatory proteins such as hepcidin. The resulting iron overload further amplifies the inflammatory response, forming a vicious cycle.

The NAR is a novel systemic inflammatory and nutritional marker that has become a promising noninvasive biomarker for NAFLD. It reflects both inflammation and liver nutritional status, and has potential utility in predicting the presence and progression of the disease. A study of 1,192 T2DM patients found that the neutrophil-to-lymphocyte ratio (NLR) was not associated with DN (41), whereas another study involving 4,813 patients with T2DM reported that the NLR was associated with DN but not with DR (42). Other studies have concluded that both NLR and platelet-to-lymphocyte ratio (PLR) are associated with DR and diabetic peripheral neuropathy (DPN) (43–46). However, the relationship between NAR, another novel inflammatory marker, and DR has not yet been studied. In our study, elevated NAR levels were positively correlated with the risk of DR, and we found that NAR mediated 20.99% of the relationship between ferritin and DR, indicating that NAR partially explains the association between ferritin and DR. Therefore, controlling inflammation can reduce the incidence of DR. Our study emphasizes that the inflammatory index NAR plays an indirect role in the relationship between ferritin and the occurrence of DR.

We also found through the ROC curve analysis that the combination of ferritin and NAR indices can better predict the risk of DR, with an AUC of 0.68. Therefore, for patients with T2DM (aged ≥ 45 years), it is essential to monitor changes in blood glucose and lipid levels, as well as changes in blood cell counts and nutritional indices. This is crucial for the early prevention of DR.

It is important to note the limitations of this study. First, the cross-sectional design precludes the establishment of a causal relationship between elevated SF levels and DR development, which requires validation in prospective studies. Secondly, the majority of DR cases in our cohort were nonproliferative diabetic retinopathy (NPDR), with a limited number of proliferative diabetic retinopathy (PDR) cases, which limited our ability to perform detailed stratified analyses. Future studies with larger sample sizes are needed to address this.

## Conclusion

The findings of this study suggest that elevated ferritin levels in middle-aged and elderly individuals with T2DM are associated with the occurrence of DR. Furthermore, combining ferritin and NAR for diagnostic purposes may serve as a sensitive marker for the early detection of DR. Moreover, inflammatory indicators such as NAR significantly mediate the risk of DR, indicating that inflammatory responses play an important role in the etiology of DR. Monitoring changes in ferritin and inflammatory markers such as NAR during the follow-up of T2DM patients is an effective approach to enhance the prevention of DR.

## Data Availability

The data analyzed in this study is subject to the following licenses/restrictions: Due to the sensitive nature of the data (e.g., patient information), access is restricted. Data are available from the corresponding author upon reasonable request and with permission from the Lanzhou University First Hospital. Requests to access these datasets should be directed to Jingfang Liu, 824168@126.com.
